# Diabetic Ketoacidosis With Schmidt Syndrome: An Autoimmune Polyendocrine Syndrome Type 2

**DOI:** 10.7759/cureus.81588

**Published:** 2025-04-01

**Authors:** Munhill Shahzad, Faheem Zaman

**Affiliations:** 1 Medicine, Ayub Teaching Hospital, Abbottabad, PAK

**Keywords:** autoimmune addison’s disease, autoimmune polyendocrine syndrome, diabetic ketoacidosis, schmidt syndrome, thyroiditis

## Abstract

Schmidt syndrome or autoimmune polyglandular syndrome type 2 is an autoimmune disorder that affects many hormone-producing (endocrine) glands. With the role of genetics and familial predisposition, autoimmune thyroid disease in combination with Addison's disease is the most common presentation. Diabetes mellitus, hyperparathyroidism, pernicious anemia, hypogonadism, vitiligo, chronic atrophic gastritis, chronic autoimmune hepatitis, alopecia, myasthenia gravis, rheumatoid arthritis, Sjögren's syndrome, and thrombocytic purpura may or may not be present.

We present a case of a 28-year-old male, already diagnosed with Addison’s disease for the past eight years. He presented to our medical department with nausea and vomiting over two days. On presentation, he was lethargic and dehydrated with sunken eyes as well as discoloration of the gums and skin. His abdomen was soft but mildly tender. He had high serum glucose levels and was diagnosed as a case of diabetic ketoacidosis based on urinary ketones and arterial blood gases, after being diagnosed with diabetes two months prior. On further investigation, his thyroid function test was deranged two months ago as well. This syndrome is a rare autoimmune disorder that is difficult to diagnose because its symptoms depend on which gland becomes involved first. The patient was treated and improved with corticosteroids, thyroxine, and insulin therapy.

## Introduction

Autoimmune polyglandular syndrome type 2 (APS type 2) was recognized and first described in 1926 by Martin Benno Schmidt, and has henceforth also been known as Schmidt syndrome. This immunoendocrine disorder involves the following three main glands: adrenals, thyroid, and pancreas. For the diagnosis, Addison's disease must occur in combination with autoimmune thyroid disease and/or type 1 diabetes mellitus [1]. Other conditions associated with this syndrome are coeliac disease, myasthenia gravis, and primary hypogonadism.

The basis of this disorder is that people diagnosed with Addison's disease are at higher risk of developing other associated autoimmune diseases than the general population. The initial presentation may include fatigue, generalized weakness, weight loss, nausea, vomiting, abdominal pain, dizziness, tachycardia, and hypotension. Addison's disease combined with Hashimoto's thyroiditis (Schmidt syndrome) is the most common clinical presentation, while the rarest combination involves Addison's disease, Graves' disease, and type 1 diabetes mellitus. According to a meta-analysis, Addison's disease occurs in 100% of patients with APS-2, autoimmune thyroid diseases in 69-82%, and type 1 diabetes mellitus in 30-52% [2].

APS-2 has been found to be more prevalent among females with a ratio of 3:1, and peak incidence has been observed in adulthood, around the second to fifth decades. Hence, middle-aged women are at the highest risk. Genetic predisposition also plays a part. It is associated with HLA-DR3 and/or HLA-DR4 haplotypes, and the pattern of inheritance, although autosomal dominant, has shown significant variance in expression. Other non-HLA genes also believed to contribute to the inheritance are cytotoxic T-lymphocyte protein 4 (CTLA-4), protein tyrosine-protein phosphatase, and non-receptor type 22 (PTPN22) [3].

About 10% of patients with APS-2 and Addison’s disease have been found to have a relative with adrenal insufficiency, and about 10% of patients with APS-2 and type 1 diabetes have a sibling with the same disease or with autoimmune thyroid disease [4]. Prevalence is estimated to be 1.4-2.0 per 100,000 population [5].

## Case presentation

**Figure 1:**
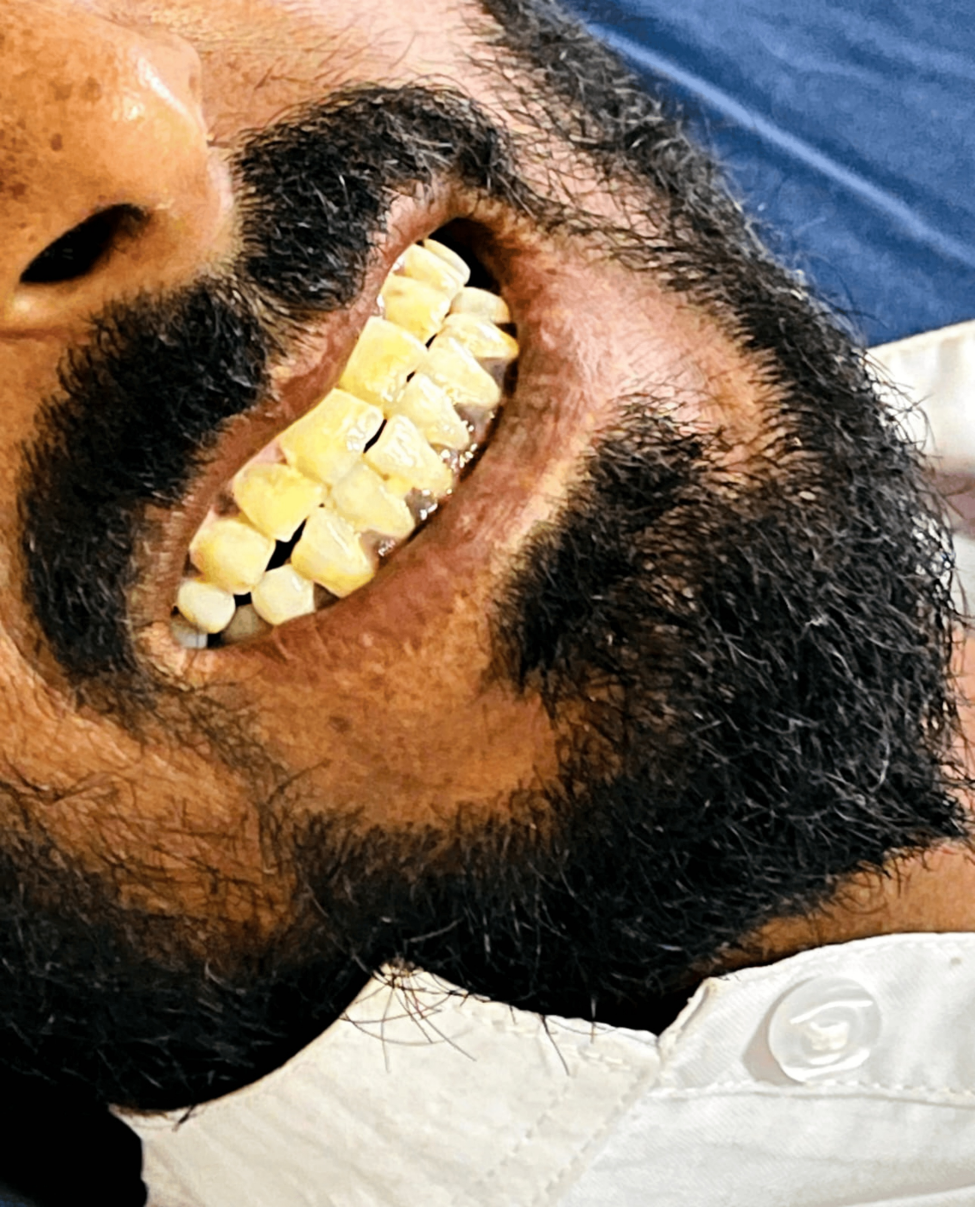
Hyperpigmentation of the gums.

**Figure 2:**
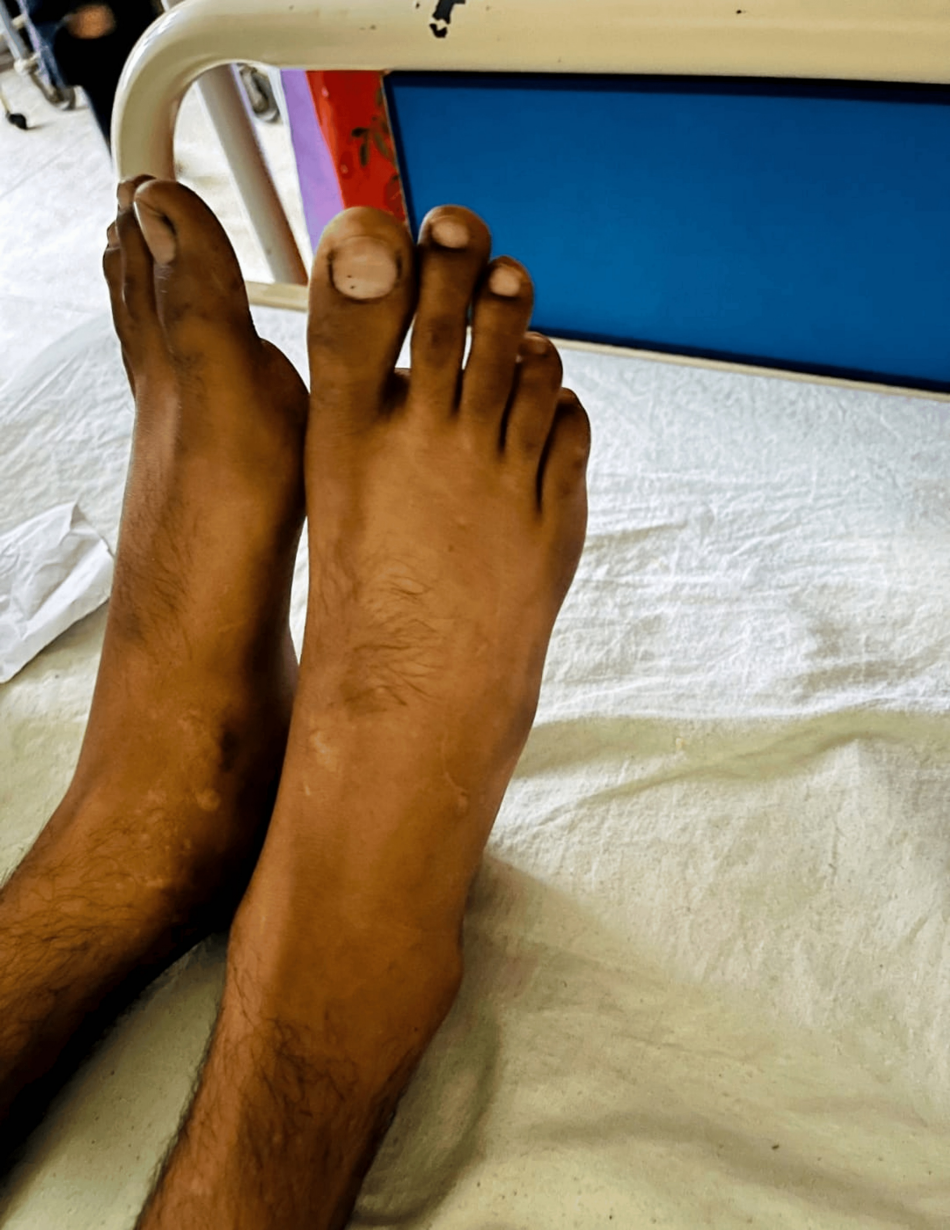
Hyperpigmentation of the feet.

**Figure 3:**
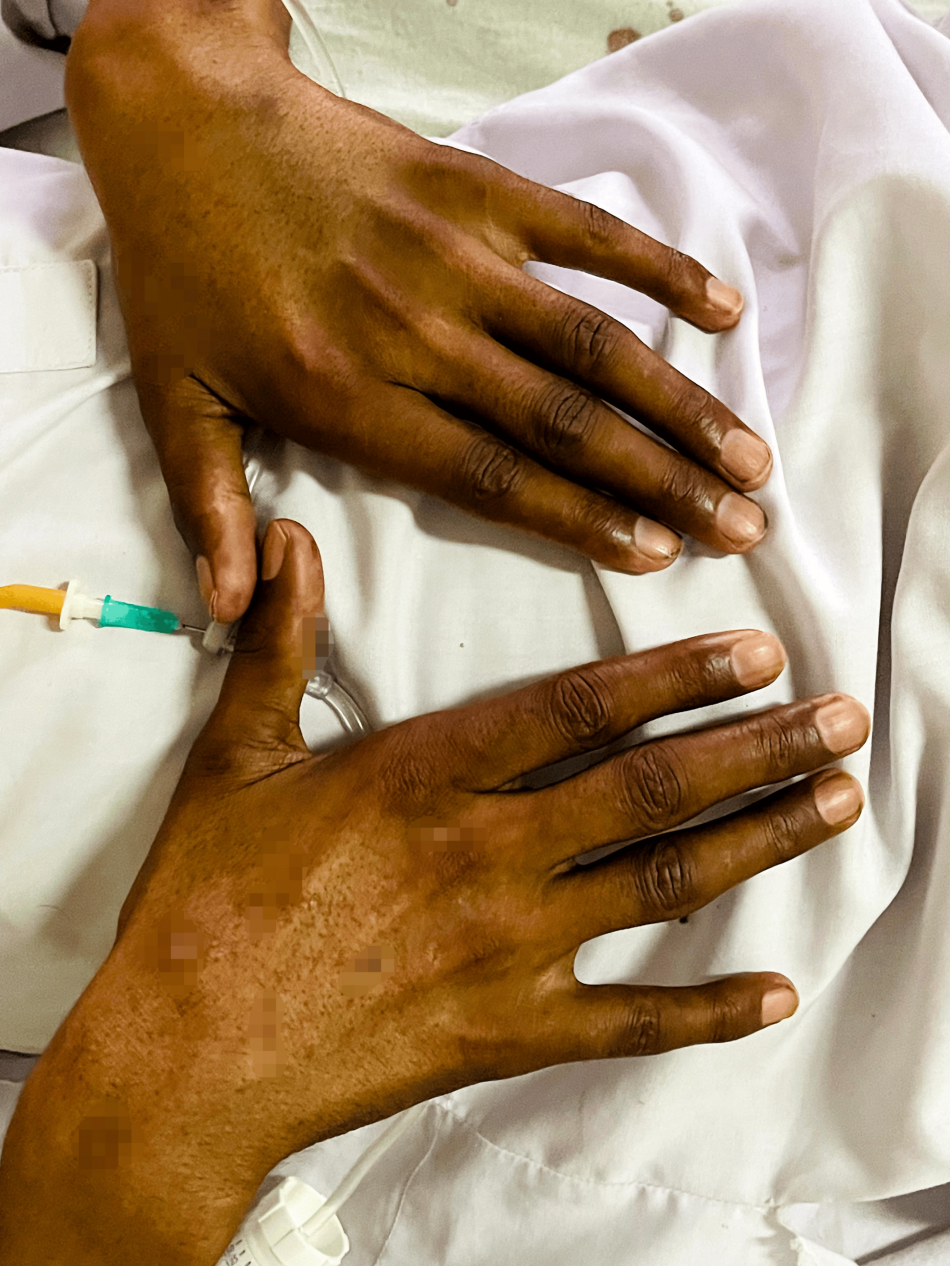
Hyperpigmentation of the hands.

We present a case of a 28-year-old male, already diagnosed with Addison’s disease for the past eight years. He presented to our medical department with nausea and vomiting over two days. On presentation, hyperpigmentation of the gums and skin of the feet and hands was immediately noted when examined (Figures 1-3). His abdomen was soft but mildly tender. Lung fields were clear bilaterally, and no added heart sounds were perceived. Although conscious and oriented, he was lethargic and dehydrated with sunken eyes. He was thus admitted to the medical unit.

Previous medical history included abdominal tuberculosis treated in 2013. Eight years prior to the presentation, he had been admitted to a tertiary care center with fatigue, postural hypotension, and tachycardia. In addition, abdominal pain with nausea, vomiting, and progressive darkening of skin was also a complaint. His serum cortisol at 8 am had been 45 ng/dL (normal range: 170-450 ng/dL), which was lower than the normal range. Therefore, he was started on fludrocortisone 0.1 mg once a day and betamethasone 0.5 mg once a day.

Two months before presenting to our unit, he was diagnosed with diabetes mellitus based on fasting blood glucose levels of 271 mg/dL, post-prandial levels in the range of 496 mg/dL, and an HbA1c of 11.6%, along with symptoms of polydipsia and polyuria. He was started on metformin 500 mg. An episode of thyrotoxicosis was also recorded to have occurred two months ago as he presented with symptoms of palpitation, sweating, increased appetite, weight loss, and fatigue. Thyroid function showed initial hyperthyroidism. He was then started on thyroxine at that time. For this, his previous medications for Addison’s disease as well as DM were continued. Family history was positive for hypertension, diabetes mellitus, and pulmonary tuberculosis.

Further inquiry revealed that he had been non-compliant with both betamethasone and fludrocortisone for over a year, as well as the medications for his diabetes. Thyroid function tests were thyroid-stimulating hormone (TSH) 1.78 (0.4-4.5) mIU/L, T3 of 0.527 (0.69-2.15) nmol/L, and T4 of 7.17 (5.2-12.789) mcg/dL. Electrolytes were serum sodium of 142 (135-155) mmol/L and potassium was 3.5 (3.5-5.5) mmol/L. Complete blood count showed TLC of 4,700/cmm (4,000-11,000/cmm), platelets of 264,000/cmm (150,000-400,000/cmm), and hemoglobin level of 14.1 (11.5-17.5) g/dL. Arterial blood gases showed a pH of 7.3 (7.35-7.45), HCO_3_ of 16.4 (21-28), and PCO_2_ of 24.7 (35-45). Urinalysis showed ketone +++ and glucose +++. The rest of the labs were unremarkable. With a glycated hemoglobin of 10.4%, an RBS of 539 mg/dL, and a urinalysis positive for glucose (+++) and ketones (+++), we started him on the diabetic ketoacidosis treatment protocol. He was initially started on intravenous insulin along with intravenous fluids, and later switched to subcutaneous insulin. Potassium chloride (KCl) infusion was started when the potassium level dropped to <3.5 mmol/L. Urinary ketone levels dropped from +++ to no ketones over time, and blood glucose dropped from 539 to 250 mg/dL, at which point he was shifted to subcutaneous insulin.

Given the simultaneous presence of adrenal, thyroid, and pancreatic involvement, we suspect the patient to be on an autoimmune spectrum, specifically autoimmune polyendocrine syndrome type 2. Initially, we believed the hyperglycemia stemmed from the steroid medication used to control the patient’s Addison’s disease and thyrotoxicosis. However, not only had the patient not been taking his prescription for over a year, but he had a very prominent history of raised glycated hemoglobin on multiple occasions. It was during this time period of non-compliance that he had been diagnosed with diabetes mellitus.

It was also observed that it was particularly difficult to normalize the patient’s blood glucose levels, and intense monitoring was required on the part of the team of doctors and nurses. He was started on regular insulin subcutaneously and then switched to insulin 70/30. Other treatments, including fludrocortisone, thyroxine, and pantoprazole in the form of tablets, were also started. Increasingly higher doses of insulin were required to revert to a euglycemic state, despite the tendency of Addisonian patients to become hypoglycemic relatively quickly.

After a single recorded episode of thyrotoxicosis, no further instances of hyperthyroidism were reported. During his admission to our facility, the patient underwent workup, which revealed normal thyroid function tests. However, we suspect the patient may be transitioning toward hypothyroidism, as his labs show a trend from hyperthyroid to euthyroid, with a potential shift to hypothyroidism in the future. A thyroid autoimmune panel was conducted, which included testing for serum anti-thyroid peroxidase (anti-TPO) and serum anti-thyroglobulin antibodies (anti-Tg). The results revealed elevated levels of both antibodies: anti-TPO >1,000 IU/L (with values ≥1.75 IU/L considered high) and anti-Tg = 346.87 IU/mL (with values ≥4.11 IU/mL considered high).

Additionally, serum TSH receptor antibody level was found to be 9.28 IU/L (with values ≥1.75 IU/L considered high), leading to decreased thyroid hormone production. The patient’s serum glucose levels improved and stabilized on insulin therapy. He was discharged on insulin injections, tablet Florinef, tablet Perzol, and tablet thyroxine.

## Discussion

Due to the involvement of multiple endocrine glands, Schmidt syndrome presents unique challenges in treatment strategies. Since management often involves the use of both glucocorticoids and insulin, these patients are at higher risk of both hypo and hyperglycemia as well as diabetic ketoacidosis, similar to how we experienced the latter in our patient. The opposing effects of insulin and glucocorticoids on glucose homeostasis need to be balanced, and the physician must know how to synchronize these two treatments [6].

Since this combination of disorders is particularly rare, resources, data, and management are equally lacking. Where other studies have shown that T1DM developed before Addison's, the present case is unique, where the patient was diagnosed with diabetes after a prolonged diagnosis of Addison's, showing the later involvement of the pancreas. Patients with DM in the context of APS-2 have a high frequency of positive ICA, GADAbs, or IA2 Abs [7]. As a follow-up, we recommend screening for these antibodies on suspicion of this combination.

A similar case was reported in 2022 when a nine-year-old male was diagnosed with APS 2. He had adrenal insufficiency three years ago due to lack of adherence to hydrocortisone. After an episode of hemodynamic instability, he was admitted to the hospital. Laboratory investigations revealed that he had Hashimoto's thyroiditis and Celiac disease. Later on, glutamic acid decarboxylase antibody (GAD) anti-islet cell antibodies were also positive, predisposing him to diabetes mellitus type 1 [8].

On the other end of the age spectrum, another case of Schmidt syndrome was a 60-year-old female patient who was a known type 1 diabetic patient, on insulin therapy for the past 20 years. On presentation, her laboratory investigations revealed a raised TSH (6 mU/mL) as well as anti-TPO antibodies (251 IU/mL), but normal free T3 and T4. Based on these labs, a diagnosis of hypothyroidism was reached, and the combination of the pre-existing diabetes mellitus type 1 and hypothyroidism led to the conclusion of Schmidt syndrome [9].

## Conclusions

It is remarkable that one out of four patients suffering from one autoimmune disease will develop another during their lifetime. For this reason, physicians must be aware of the tendency of autoimmune diseases to cluster within the same individual. Therefore, a high level of diagnostic suspicion for Schmidt syndrome is required, especially when encountering patients with a monoglandular autoimmune disease and atypical somatic or psychological complaints. Furthermore, screening of organ-specific autoantibodies in these patients could help in the detection of those at risk of developing Schmidt syndrome. This is also important because the initiation of thyroid hormone in a patient with autoimmune thyroiditis and misdiagnosed adrenal insufficiency may precipitate an acute adrenal crisis, which happens because thyroxine stimulates increased hepatic metabolism of corticosteroids.
